# Comparison of lateral entry with crossed entry pinning for pediatric supracondylar humeral fractures: a meta-analysis

**DOI:** 10.1186/s13018-018-0768-3

**Published:** 2018-04-03

**Authors:** Yuyan Na, Rui Bai, Zhenqun Zhao, Changxu Han, Lingyue Kong, Yizhong Ren, Wanlin Liu

**Affiliations:** 1 Department of Arthroscopy and Sports Medicine, The Second Affiliated Hospital of Inner Mongolia Medical University, Hohhot, 010030 Inner Mongolia Autonomous Region China; 2 Department of Pediatric Orthopedics, The Second Affiliated Hospital of Inner Mongolia Medical University, Hohhot, 010030 Inner Mongolia Autonomous Region China

**Keywords:** Supracondylar humeral fracture, Pediatric, Pin fixation

## Abstract

**Background:**

The standard treatment for severe displaced pediatric supracondylar humeral fracture (SCHF) is closed reduction and percutaneous pin fixation. However, controversy persists concerning the optimal pin fixation technique. The purpose of this study was to compare the safety and efficacy on the configuration of lateral entry only with crossed entry pin fixation for SCHF, including Gartland type II and type III fractures in children.

**Methods:**

Published literatures, including retrospective studies, prospective studies, and randomized controlled trials, presenting the probability of poor functional consequence of elbow and/or loss of reduction and/or iatrogenic ulnar nerve injury and/or superficial infection and/or cubitus varus were included. Statistical analysis was performed with the Review Manager 5.3 software.

**Results:**

Twenty-four studies were included in the present meta-analysis involving 1163 patients with lateral entry pins and 1059 patients with crossed entry pins. An excellent score of Flynn criteria occurred more commonly in patients who treated with crossed pins than in patients with lateral pins only (RR = 0.93; 95% CI 0.87–0.99; *P* = 0.03). In accordance with previous systematic review, the incidence of iatrogenic ulnar nerve injury in crossed entry group was significantly more than in lateral entry group with statistical difference (RR = 0.26; 95% CI 0.14–0.47; *P* < 0.0001). And, results of subgroup analysis on iatrogenic ulnar nerve injury based on experimental design of retrospective study (RR = 0.23; 95% CI 0.10–0.52; *P* < 0.0004) and randomized control trial (RR = 0.29; 95% CI 0.10–0.79; *P* < 0.02) were similar.

**Conclusions:**

In consideration of the contradictoriness of lateral entry with two pins only (possible risk of poor functional consequence of elbow) and crossed entry pins (risk of iatrogenic ulnar nerve injury), the recommended strategy for the treatment of SCHF is the lateral entry technique with introducing divergent three pins which can provide a stable configuration and avoid the injury of the ulnar nerve. And additional protective measures for the ulnar nerve should be taken by surgeons that wish for the more stable structure with the crossed entry technique.

## Background

Supracondylar humeral fracture is the most common type of elbow fracture in children younger than 15 years [1]. Children are susceptible to this fracture by reasons of the bending structure and the weak metaphyseal sclerotin of the distal humerus as well as the thin ridge of metaphyseal bone between the coronoid fossa and the olecranon fossa. The fracture is classified, most commonly, according to Gartland’s criteria as the Gartland type I fracture is stable and not displaced, and varying degrees of displacement and angulation are present in Gartland type II and III fracture [2].

The impact transmitted to the outstretched wrist causes the elbow to hyperextend when falls lead the olecranon to gather most of the impact at the humeral supracondylar and the axial force is converted to a bending force at this region, resulting in the extension-type supracondylar humeral fracture. And a fall on the olecranon with elbow flexion leads to the flexion-type supracondylar fracture. It has been reported that 98% of the patients with the supracondylar humeral fracture (SCHF) are extension-type fracture in Chinese children [3]. Cast fixation is a mainstream way to prevent the displacement of fracture segments for Gartland type I [4]. However, agreement has not been reached on the pinning technique and configuration after closed reduction for severely displaced Gartland type II and type III fractures [5]. There are two common techniques of pin fixation: lateral entry pins only and crossed entry pins with at least one medial and one lateral [6]. Theoretically, crossed entry pins have the advantage of enhanced mechanical stability of the configuration, yet this technique increases the potential injury of the ulnar nerve [7, 8]. And lateral entry pins only may reduce mechanical stability of the structure, although ulnar nerve injury can be avoided [9, 10].

In view of the respective advantages of the two pinning techniques, we carried out a review on the published literature to compare the safety and efficacy on the configuration of lateral entry only with crossed entry pin fixation for SCHF, including Gartland type II and type III fractures in children. The contents of comparisons include ulnar nerve injury caused by pin placement, loss of reduction according to the radiographic outcomes, elbow functional outcomes assessed by Flynn criteria, and short-term complication, such as superficial infection, as well as long-term complication, for instance, cubitus varus.

## Methods

### Search strategy

Published literatures, including retrospective studies, prospective studies, and randomized controlled trials, were searched without any ethnicity and language restriction in the electronic databases PubMed, Google Scholar, and Chinese National Knowledge Infrastructure (CNKI) based on the following MeSH terms: “supracondylar fracture,” “humeral,” “kirschner pins,” and “child or pediatric.” Besides, references of all included articles were also reviewed. The retrieved articles had to be published as a full text, and the last search for these studies was up to May 31, 2017.

### Inclusion/exclusion criteria

Eligible studies in the present meta-analysis were selected according to the following criteria: (1) comparative studies on pinning technique for SCHF with crossed entry and isolated lateral entry; (2) patients of the included studies should be treated with percutaneous pinning after closed reduction, yet a few with a small incision to protect the ulnar nerve were also included; and (3) only Gartland type II and type III fractures with percutaneous pin fixation were included. Studies that contained nonoperative treatment or Gartland type I fracture were excluded.

### Data extraction

The following information was extracted from the eligible studies independently by two authors in our team: the first author’s name, year of publication, Gartland type for SCHF, size of lateral entry group and crossed entry group, and average age of patients as well as research design of the included studies.

### Statistical analysis

Statistical analysis was performed with the Review Manager 5.3 software. To evaluate safety and efficacy between lateral entry group and crossed entry group, the relative risks (RR) and 95% confidence intervals (CI) for ulnar nerve injury, loss of reduction, Flynn criteria on elbow functional outcomes, and superficial infection as well as cubitus varus were calculated. The heterogeneity among the included studies were estimated using the chi-squared test and *I*^2^ test. If the corresponding *P* > 0.05 or *I*^2^ < 50%, which was considered less heterogeneity among these studies, then the fixed effects model was used to calculate the pooled RRs; otherwise, the random-effects model was applied. And publication bias among these studies in each comparison were assessed by the symmetric construction of a funnel plot.

## Results

### Search results and study characteristics

Using our search strategy resulted in the identification of 1125 relevant articles initially. After excluding duplications and screening their titles and abstracts according to the inclusion and exclusion criteria, 24 studies were included in the present meta-analysis involving 1163 patients with lateral entry pins and 1059 patients with crossed entry pins. Of the 24 included studies, 9 were randomized control trials [11–19], 5 were prospective studies [20–24], and 10 were retrospective studies [25–34]. And the characteristics of the included studies are listed in Table 1.

**Table 1:** Clinical characteristics of included studies

Study	Year	No. of patients		Mean age (years)		Fracture type	Design
		LE	MLE	LE	MLE		
Xiang et al.	2017	33	32	6.4	6.5	Gartland III	Retrospective study
Chen et al.	2017	39	39	7.1	7.5	Gartland II and III	Randomized control trial
Zeng et al.	2017	18	16	7.9	8.4	Gartland III	Retrospective study
Tao et al.	2016	92	104	9.6 ± 3.0	9.8 ± 2.8	Gartland III	Retrospective study
Zhang et al.	2014	62	86	5.7 ± 2.8	6.3 ± 3.0	Gartland II and III	Retrospective study
Zhong et al.	2009	45	72	5.6	6.8	Gartland II and III	Retrospective study
Kocher et al.	2007	28	24	6.1 ± 1.5	5.7 ± 1.6	Gartland III	Randomized control trial
Foead et al.	2004	27	28	5.8		Gartland II and III	Randomized control trial
Tripuraneni et al.	2009	20	20	4.3	5.5	Gartland II and III	Randomized control trial
Gaston et al.	2010	47	57	5.7	6.2	Gartland III	Randomized control trial
Maity et al.	2012	80	80	6.1 ± 1.8	6.2 ± 1.8	Gartland II and III	Randomized control trial
Vaidya et al.	2009	29	31	5.8	6.2	Gartland III	Randomized control trial
Anwar et al.	2011	25	25	7.0		Gartland II and III	Randomized control trial
Topping et al.	1995	20	27	6.1	7.3	Gartland III	Retrospective study
Devkota et al.	2008	23	79	7.8		Gartland III	Prospective study
Khan et al.	2007	14	31	8.1		Gartland II and III	Prospective study
Kwak-Lee et al.	2014	244	47	4.5	5.4	Gartland II and III	Prospective study
Mazda et al.	2001	82	26	5.6		Gartland II and III	Prospective study
Singh et al.	2013	17	15	7.1 ± 3.2	7.9 ± 3.3	Gartland II and III	Prospective study
Mahmood et al.	2013	30	30	6.0	6.0	Gartland III	Retrospective study
Prashant et al.	2016	31	31	8.3	8.6	Gartland III	Randomized control trial
Reisoglu et al.	2016	48	39	6.2	6.1	Gartland III	Retrospective study
Sahu et al.	2013	85	85	7.8		Gartland III	Retrospective study
Solak et al.	2003	24	35	5.0		Gartland III	Retrospective study

*LE* lateral entry, *MLE* medial and lateral entry

### Elbow functional outcomes of percutaneous pinning

**Fig. 1:**
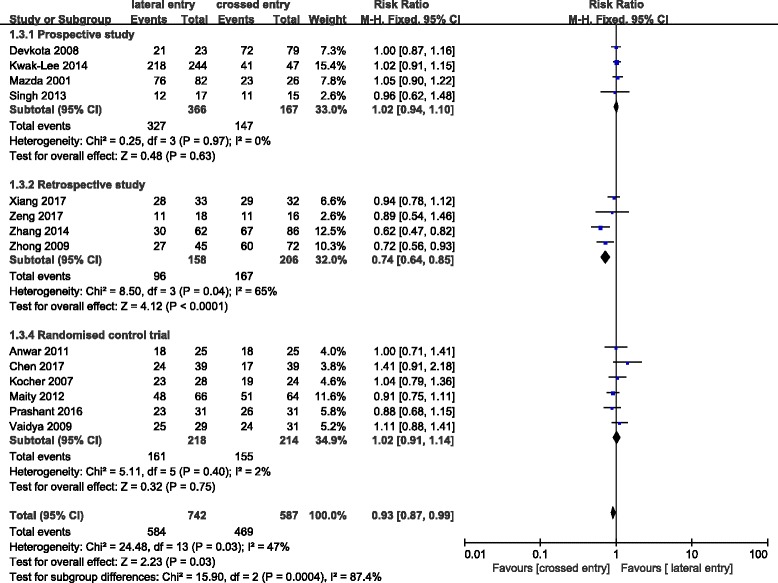
Comparison of elbow functional outcomes between the lateral entry group and the crossed entry group

Flynn criteria of elbow were reported in 14 studies which are based on the elbow motion and carrying angle [35]. An excellent score was considered acceptable when at final follow-up evaluation. We identified a significant difference in Flynn criteria between the lateral entry group and the crossed entry group. An excellent score occurred more commonly in patients who are treated with crossed medial and lateral pins than in patients with lateral pins only (RR = 0.93; 95% CI 0.87–0.99; *P* = 0.03) (Fig. 1).

### Radiographic outcomes

**Fig. 2:**
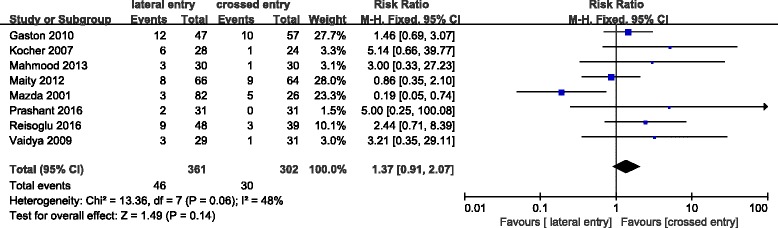
Comparison of loss of reduction through imaging between the lateral entry group and the crossed entry group

Loss of reduction were pointed out in nine studies which are evaluated on the basis of the change in the Baumann angle. No displacement (change in the Baumann angle of < 6° on the anteroposterior radiograph) was considered acceptable by Skaggs et al. [36, 37]. Loss of reduction (mild and major displacement) occurred in 46 (12.7%) of 361 patients treated with lateral pins and in 30 (9.9%) of 302 patients treated with crossed pins, however, which did not reach critical value of statistical difference (*P* = 0.14) (Fig. 2).

### Short-term complications

**Fig. 3:**
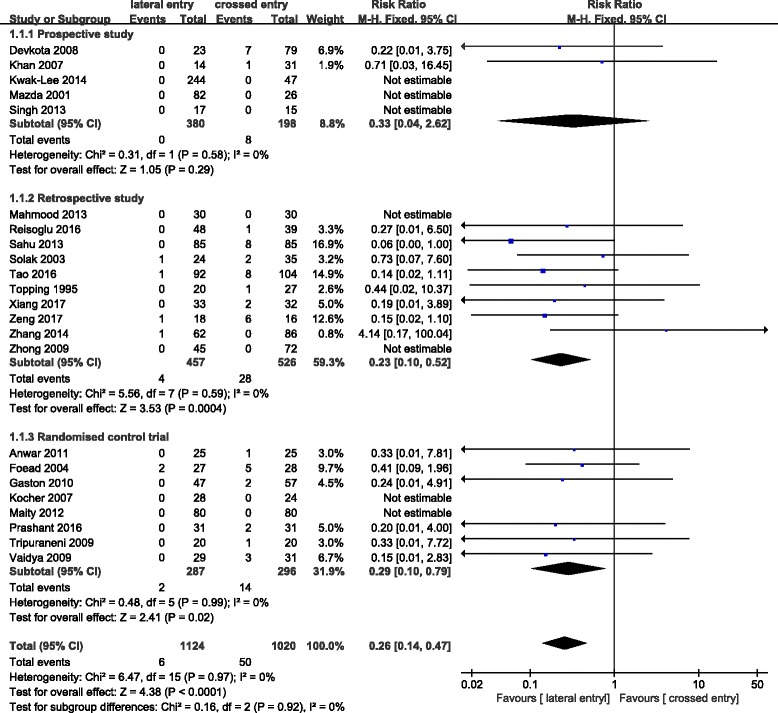
Comparison of iatrogenic ulnar nerve injury between the lateral entry group and the crossed entry group

**Fig. 4:**
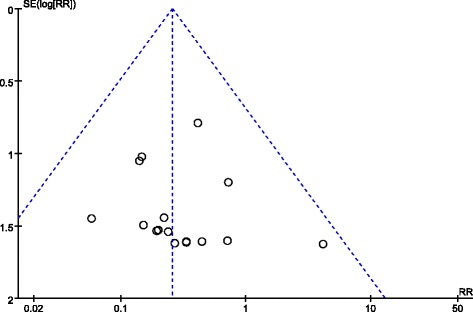
Funnel plot for iatrogenic ulnar nerve injury to detect publication bias

**Fig. 5:**
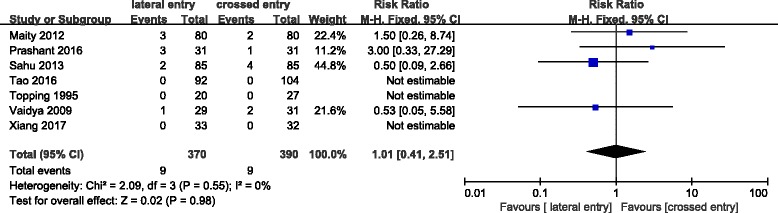
Comparison of superficial infection between the lateral entry group and the crossed entry group

Short-term complications, such as iatrogenic ulnar nerve injury and superficial infection, were compared in two groups of population. Iatrogenic ulnar nerve injury occurred in 6 (0.5%) of 1124 patients treated with lateral pins and in 50 (4.9%) of 1020 patients treated with crossed pins. The incidence of ulnar nerve injury in the crossed entry group was significantly more than that in the lateral entry group with statistical difference (RR = 0.26; 95% CI 0.14–0.47; *P* < 0.0001) (Fig. 3). Heterogeneity determination suggested that no obvious heterogeneity existed in the present pooled analysis (*P* = 0.97; *I*^2^ = 0%). Publication bias on iatrogenic ulnar nerve injury was assessed by funnel plot which shows no obvious asymmetry exist (Fig. 4). Results of subgroup analysis on iatrogenic ulnar nerve injury based on experimental design of retrospective study (RR = 0.23; 95% CI 0.10–0.52; *P* < 0.0004) and randomized control trial (RR = 0.29; 95% CI 0.10–0.79; *P* < 0.02) were similar (Fig. 3). However, the analysis result of the prospective study was completely different which may be due to the limited number of patients with iatrogenic ulnar nerve injury. No significant difference between the two groups was observed in terms of superficial infection (Fig. 5). And no statistical heterogeneity was detected in the pooled results on superficial infection.

### Long-term complication

**Fig. 6:**
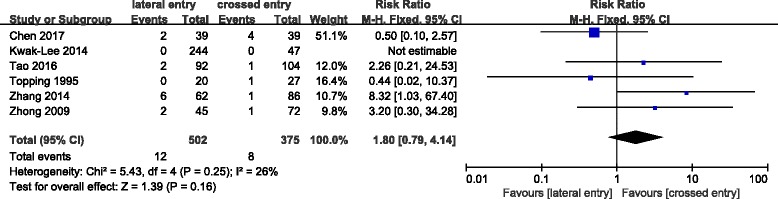
Comparison of cubitus varus between the lateral entry group and the crossed entry group

Six studies investigated the relationship between pin fixation techniques and cubitus varus. Incidence of this complication was low either in the lateral entry group or in the crossed entry group with 2.3 and 2.1%, respectively. After comparison of the two fixation methods, no significant difference was identified in terms of cubitus varus (Fig. 6).

## Discussion

The two major complications for SCHF treatment debated are risk of ulnar nerve injury and stable configuration with lower risk of residual deformity and excellent functional outcome. In our present results, there was a significant trend toward getting better functional consequence of elbow with crossed entry pins instead of lateral entry only. However, more orthopedic surgeons prefer to choose lateral pinning technique than crossed pinning due to the increased risk of iatrogenic ulnar nerve injury which was confirmed by our meta-analysis results. Additionally, the risks of loss of reduction in radiograph or developing late deformity, such as cubitus varus, were similar for both pinning groups.

The analysis result revealed that iatrogenic ulnar nerve injury mainly occurred in the crossed entry group which was in accordance with previous systematic review [5, 38–40]. Brauer and colleagues believed that the probability of iatrogenic ulnar nerve injury with medial/lateral entry pins is 5.04 times higher than that with lateral entry pins in their systematic review which includes randomized control trial, prospective, and retrospective study [38]. Woratanarat et al. suggested that lateral entry technique is preferable to crossed pinning technique for SCHF fixation as a result of decreased risk of iatrogenic ulnar nerve injury [40]. And Zhao et al. analyzed ulnar nerve injury by reviewing seven randomized control trials and found that nerve injury was higher with medial/lateral entry pins than with lateral entry pins (3.33 times) [5]. In the subgroup analysis based on experimental design, the probability of ulnar nerve injury in the randomized control trial group was in line with that in Zhao et al. with 3.45 times. However, most included studies did not note the corresponding treatment to ulnar nerve injury which makes the further analysis of optimal therapy difficult to carry out.

Few reviews evaluated postoperative functional outcomes between the two treatment techniques on account of the related content in clinical study which is limited. Zhao et al. did not observe significant difference between the two fixations in terms of functional outcomes in three randomized control trials which was in accordance with our present subgroup analysis result based on experimental design of randomized control trials (Fig. 1). However, the total result suggested that better functional consequence of elbow, including elbow motion and carrying angle, occurred more commonly in crossed entry group than in crossed entry group. Due to the confounding bias of retrospective studies, the total result must be interpreted with caution and confirmed by reviews with lager sample size and preferable experimental design.

There are several limitations in our present analysis that need to be addressed. First, ten studies included in our meta-analysis are retrospective reports with the potential for numerous confounding bias which may provide weaker evidence for evaluation of safety and efficacy on the configuration of a lateral entry only or crossed pinning entry for SCHF. Therefore, subgroup analysis on iatrogenic ulnar nerve injury and functional consequence of elbow based on experimental design were performed. Second, the surgical technique of percutaneous pinning varies within included studies which may weaken the power of those comparisons. Third, several clinical characteristics and interventions, such as fracture type (including Gartland II/III), average follow-up period, and number of pins, were inconsistent within included studies.

In consideration of the contradictoriness of lateral entry with two pins only (risk of unsatisfactory outcome of treatment based on elbow motion and carrying angle, although it must be interpreted with caution due to the possible confounding bias of retrospective studies) and crossed entry pins (risk of iatrogenic ulnar nerve injury), the recommended strategy for the treatment of SCHF is the lateral entry technique with introducing divergent three pins which can provide a stable configuration and avoid the injury of ulnar nerve. And additional protective measures for the ulnar nerve should be taken by surgeons that wish for the more stable structure with the crossed entry technique, such as palpating the ulnar nerve and pushing it posteriorly with the thumb or making a small incision over the medial epicondyle and inserting pin under direct visualization.

## Conclusion

In conclusion, crossed entry pins for the treatment of SCHF suffer from a higher risk of iatrogenic ulnar nerve injury than lateral entry pins. And lateral entry with two pins only may have a higher risk of poor functional consequence of elbow than crossed entry pins; besides, no difference was found when compared with crossed entry pins. Therefore, the recommended strategy for the treatment of SCHF is the lateral entry technique with introducing divergent three pins which can provide a stable configuration and avoid the injury of ulnar nerve.

## Abbreviations

CI: Confidence intervals
CNKI: Chinese National Knowledge Infrastructure
RR: Relative risks
SCHF: Supracondylar humeral fracture
